# Aqua­[1,8-bis­(pyridin-2-yl)-3,6-dithia­octane-κ^4^ *N*,*S*,*S*′,*N*′]copper(II) dinitrate acetonitrile monosolvate

**DOI:** 10.1107/S1600536811056145

**Published:** 2012-01-11

**Authors:** Mayra Manzanera-Estrada, Marcos Flores-Alamo, Jean-Michel Grevy M., Lena Ruiz-Azuara, Luis Ortiz-Frade

**Affiliations:** a Centro de Investigacíon y Desarrollo Tecnológico en Electroquímica, Pedro Escobedo, Querétaro 76703, Mexico; b Facultad de Química, Universidad Nacional Autónoma de México, México 04510, DF, Mexico; c Centro de Investigaciones Químicas, Universidad Autónoma del Estado de Morelos, Av. Universidad 1001, Col. Chamilpa Cuernavaca, Morelos 62210, Mexico

## Abstract

In the title compound, [Cu(C_16_H_20_N_2_S_2_)(H_2_O)](NO_3_)_2_·CH_3_CN, the Cu^II^ atom displays a distorted square-pyramidal coordination, in which a water mol­ecule occupies the apical position and the basal plane is formed by two N atoms and two S atoms of a 1,8-bis­(pyridin-2-yl)-3,6-dithia­octane ligand. The crystal packing is stabilized by O—H⋯O and C—H⋯O hydrogen bonds.

## Related literature

For a related compound, see: Rodríguez-Torres *et al.* (2009 ▶). For related structures of Cu(II) complexes with 1,8-bis­(pyridin-2-yl)-3,6-dithia­octane ligands, see: Brubaker *et al.* (1979 ▶); Humphery *et al.* (1988 ▶). For a description of the geometry of complexes with five-coordinate Cu^II^ ions, see: Addison *et al.* (1984 ▶).
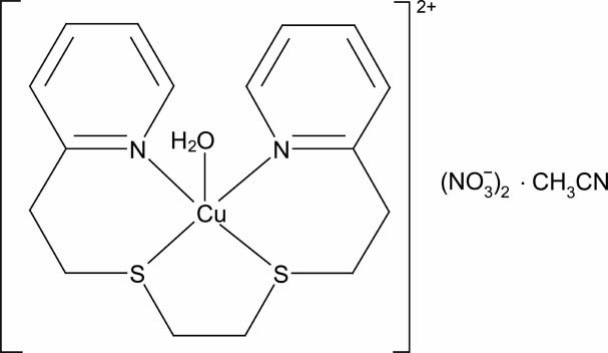


## Experimental

### 

#### Crystal data

[Cu(C_16_H_20_N_2_S_2_)(H_2_O)](NO_3_)_2_·C_2_H_3_N
*M* *_r_* = 551.09
Triclinic, 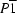

*a* = 8.8409 (5) Å
*b* = 10.8140 (5) Å
*c* = 13.5141 (6) Å
α = 79.895 (4)°
β = 71.500 (4)°
γ = 69.817 (4)°
*V* = 1146.86 (10) Å^3^
*Z* = 2
Mo *K*α radiation
μ = 1.18 mm^−1^
*T* = 138 K
0.59 × 0.30 × 0.08 mm

#### Data collection

Oxford Diffraction Gemini Atlas diffractometer
Absorption correction: multi-scan (*CrysAlis RED*; Oxford Diffraction, 2006 ▶) *T*_min_ = 0.624, *T*_max_ = 0.914
8086 measured reflections
4509 independent reflections
3744 reflections with *I* > 2σ(*I*)
*R*_int_ = 0.023

#### Refinement

*R*[*F* ^2^ > 2σ(*F* ^2^)] = 0.030
*wR*(*F* ^2^) = 0.076
*S* = 1.06
4509 reflections
305 parameters
H atoms treated by a mixture of independent and constrained refinement
Δρ_max_ = 0.66 e Å^−3^
Δρ_min_ = −0.42 e Å^−3^

### 

Data collection: *CrysAlis CCD* (Oxford Diffraction, 2006 ▶); cell refinement: *CrysAlis RED* (Oxford Diffraction, 2006 ▶); data reduction: *CrysAlis RED*; program(s) used to solve structure: *SHELXS97* (Sheldrick, 2008 ▶); program(s) used to refine structure: *SHELXL97* (Sheldrick, 2008 ▶); molecular graphics: *ORTEP-3* (Farrugia, 1997 ▶); software used to prepare material for publication: *WinGX* (Farrugia, 1999 ▶).

## Supplementary Material

Crystal structure: contains datablock(s) I, global. DOI: 10.1107/S1600536811056145/hy2502sup1.cif

Structure factors: contains datablock(s) I. DOI: 10.1107/S1600536811056145/hy2502Isup2.hkl

Additional supplementary materials:  crystallographic information; 3D view; checkCIF report

## Figures and Tables

**Table 1:** Hydrogen-bond geometry (Å, °)

*D*—H⋯*A*	*D*—H	H⋯*A*	*D*⋯*A*	*D*—H⋯*A*
O1*W*—H1*D*⋯O5^i^	0.74 (3)	1.94 (3)	2.669 (2)	169 (3)
O1*W*—H1*E*⋯O1	0.77 (2)	2.00 (3)	2.754 (3)	166 (2)
C3—H3⋯O2^ii^	0.95	2.55	3.487 (3)	168
C8—H8*A*⋯O1^iii^	0.99	2.54	3.447 (3)	152
C8—H8*B*⋯O4^iii^	0.99	2.55	3.341 (3)	137
C10—H10*A*⋯O1^iii^	0.99	2.43	3.228 (3)	137
C10—H10*B*⋯O5^iv^	0.99	2.45	3.271 (3)	140
C13—H13⋯O6^iv^	0.95	2.42	3.277 (3)	149
C14—H14⋯O2^v^	0.95	2.56	3.220 (3)	127
C16—H16⋯O6^v^	0.95	2.36	3.126 (3)	137
C17—H17*A*⋯O6	0.98	2.26	3.145 (3)	150

Symmetry codes: (i) 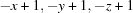; (ii) 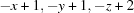; (iii) 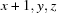; (iv) 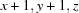; (v) 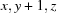.

**Fig. 1.**
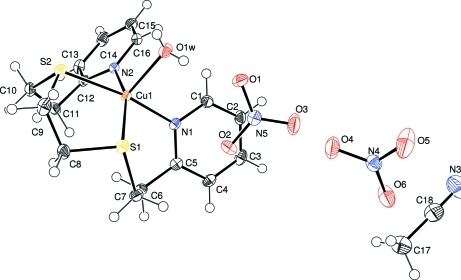
The molecular structure for the title compound. Displacement ellipsoids are drawn at the 50% probability level.

## supplementary crystallographic information

### Comment

Cu(II)–[1,8-bis(pyridin-2-yl)-3,6-dithiaoctane] complex has demonstrated biological activity against human tumor cervix line HeLa, which can be related to bio-mimetic Cu-SOD activity. Electrochemical studies indicate that the high flexibility of the 1,8-bis(pyridin-2-yl)-3,6-dithiaoctane ligand towards the preferential geometry of central atom could be an important factor in biological activity. However, the crystal structure of this compound was not obtained (Rodríguez-Torres *et al.*, 2009).

The asymmetric unit of the title compound contains one complex cation [Cu(pdto)(H_2_O)]^2+^ [pdto = 1,8-bis(pyridin-2-yl)-3,6-dithiaoctane], two nitrate anions and one acetonitrile solvent molecule (Fig. 1). The complex cation consists of a five-coordinated Cu^II^ ion in a distorted squared-pyramidal environment. The basal sites are occupied by N1, N2, S1 and S2 of the 1,8-bis(pyridin-2-yl)-3,6-dithiaoctane ligand (Humphery *et al.*, 1988). The basal Cu—N/S bond lengths are in a range of 2.0169 (17)–2.3488 (6) Å. The aqua ligand in the apical position has a Cu1—O1W bond distance of 2.1342 (16) Å, 0.129 Å shorter than that of 2.263 Å observed in [Cu(pdto)(ClO_4_)]ClO_4_ (Brubaker *et al.*, 1979). The basal CuN_2_S_2_ plane presents a slight distortion from planarity (τ = 0.1621) (Addison *et al.*, 1984), as shown by the displacements of the atoms from a mean plane through them; the metal ion is situated 0.2260 (5) Å above the N1/N2/S1/S2 plane [least-squares plane: 7.663 (2)*x* + 1.291 (4)*y* + 9.420 (4)*z* = 14.302 (4)].

The nitrate anions and acetonitrile molecule are not involved in the coordination sphere of the Cu ion. In the crystal, O—H···O and weak C—H···O hydrogen bonds stabilize the crystal packing (Table 1). The water molecule (O1W) interacts with O1 and O5 acceptor atoms of the nitrate anions, forming a *C*_2_^2^(5) motif.

### Experimental

Cu(NO_3_).2.5H_2_O (0.129 g, 0.56 mmol) was dissolved in 20 ml of anhydrous acetonitrile, followed by slow addition of 1,8-bis(pyridin-2-yl)-3,6-dithiaoctane (0.1693 g, 0.56 mmol) contained in 5 ml of anhydrous acetonitrile. A deep blue solution was obtained, and by slow ether diffusion, crystals suitable for X-ray analysis were obtained after 3 days.

### Refinement

H atoms bonded to O atom were located in difference Fourier maps and refined with *U*_iso_(H) = *U*_eq_(O). H atoms attached to C atoms were positioned geometrically and refined as riding on their parent atoms, with C—H = 0.95 (aromatic), 0.98 (methyl) and 0.99 (methylene) Å and with *U*_iso_(H) = 1.2(1.5 for methyl)*U*_eq_(C).

### Crystal data

**Table:**

[Cu(C_16_H_20_N_2_S_2_)(H_2_O)](NO_3_)_2_·C_2_H_3_N	*Z* = 2
*M**_r_* = 551.09	*F*(000) = 570
Triclinic, *P*1	*D*_x_ = 1.596 Mg m^−^^3^
*a* = 8.8409 (5) Å	Mo *K*α radiation, λ = 0.71073 Å
*b* = 10.8140 (5) Å	Cell parameters from 5580 reflections
*c* = 13.5141 (6) Å	θ = 3.5–26.0°
α = 79.895 (4)°	µ = 1.18 mm^−^^1^
β = 71.500 (4)°	*T* = 138 K
γ = 69.817 (4)°	Lamina, dark-blue
*V* = 1146.86 (10) Å^3^	0.59 × 0.30 × 0.08 mm

### Data collection

**Table:**

Oxford Diffraction Gemini Atlas diffractometer	4509 independent reflections
graphite	3744 reflections with *I* > 2σ(*I*)
Detector resolution: 10.4685 pixels mm^-1^	*R*_int_ = 0.023
ω scans	θ_max_ = 26.1°, θ_min_ = 3.5°
Absorption correction: multi-scan (*CrysAlis RED*; Oxford Diffraction, 2006)	*h* = −10→9
*T*_min_ = 0.624, *T*_max_ = 0.914	*k* = −13→12
8086 measured reflections	*l* = −15→16

### Refinement

**Table:**

Refinement on *F*^2^	Primary atom site location: structure-invariant direct methods
Least-squares matrix: full	Secondary atom site location: difference Fourier map
*R*[*F*^2^ > 2σ(*F*^2^)] = 0.030	Hydrogen site location: inferred from neighbouring sites
*wR*(*F*^2^) = 0.076	H atoms treated by a mixture of independent and constrained refinement
*S* = 1.06	*w* = 1/[σ^2^(*F*_o_^2^) + (0.0411*P*)^2^] where *P* = (*F*_o_^2^ + 2*F*_c_^2^)/3
4509 reflections	(Δ/σ)_max_ = 0.001
305 parameters	Δρ_max_ = 0.66 e Å^−^^3^
0 restraints	Δρ_min_ = −0.42 e Å^−^^3^

### Special details

**Table:**

Geometry. All e.s.d.'s (except the e.s.d. in the dihedral angle between two l.s. planes) are estimated using the full covariance matrix. The cell e.s.d.'s are taken into account individually in the estimation of e.s.d.'s in distances, angles and torsion angles; correlations between e.s.d.'s in cell parameters are only used when they are defined by crystal symmetry. An approximate (isotropic) treatment of cell e.s.d.'s is used for estimating e.s.d.'s involving l.s. planes.
Refinement. Refinement of *F*^2^ against ALL reflections. The weighted *R*-factor *wR* and goodness of fit *S* are based on *F*^2^, conventional *R*-factors *R* are based on *F*, with *F* set to zero for negative *F*^2^. The threshold expression of *F*^2^ > σ(*F*^2^) is used only for calculating *R*-factors(gt) *etc*. and is not relevant to the choice of reflections for refinement. *R*-factors based on *F*^2^ are statistically about twice as large as those based on *F*, and *R*- factors based on ALL data will be even larger.

### Fractional atomic coordinates and isotropic or equivalent isotropic displacement parameters (Å^2^)

**Table:**

	*x*	*y*	*z*	*U*_iso_*/*U*_eq_	
C1	0.5834 (3)	0.7615 (2)	0.83597 (16)	0.0179 (5)	
H1	0.5363	0.8284	0.7887	0.021*	
C2	0.4779 (3)	0.7292 (2)	0.92882 (17)	0.0204 (5)	
H2	0.3603	0.7723	0.945	0.024*	
C3	0.5462 (3)	0.6329 (2)	0.99811 (17)	0.0218 (5)	
H3	0.477	0.6107	1.0638	0.026*	
C4	0.7164 (3)	0.5699 (2)	0.97001 (16)	0.0191 (5)	
H4	0.765	0.5021	1.0161	0.023*	
C5	0.8173 (3)	0.6045 (2)	0.87531 (16)	0.0158 (4)	
C6	1.0013 (3)	0.5327 (2)	0.84063 (16)	0.0174 (5)	
H6A	1.0365	0.4776	0.901	0.021*	
H6B	1.0633	0.5981	0.8174	0.021*	
C7	1.0492 (3)	0.4443 (2)	0.75161 (16)	0.0192 (5)	
H7A	0.9807	0.3835	0.7731	0.023*	
H7B	1.1683	0.3898	0.7403	0.023*	
C8	1.2318 (3)	0.5431 (2)	0.56171 (17)	0.0199 (5)	
H8A	1.2805	0.5627	0.6115	0.024*	
H8B	1.3047	0.4573	0.5331	0.024*	
C9	1.2209 (3)	0.6514 (2)	0.47372 (17)	0.0219 (5)	
H9A	1.1771	0.6282	0.4229	0.026*	
H9B	1.3349	0.6567	0.4367	0.026*	
C10	1.2157 (3)	0.8567 (2)	0.58109 (16)	0.0181 (5)	
H10A	1.3303	0.7931	0.5631	0.022*	
H10B	1.2242	0.9452	0.5509	0.022*	
C11	1.1507 (3)	0.8592 (2)	0.70013 (16)	0.0175 (5)	
H11A	1.1456	0.7703	0.7313	0.021*	
H11B	1.2298	0.8815	0.7266	0.021*	
C12	0.9796 (3)	0.9581 (2)	0.73364 (15)	0.0156 (4)	
C13	0.9532 (3)	1.0732 (2)	0.77730 (17)	0.0200 (5)	
H13	1.0416	1.087	0.795	0.024*	
C14	0.7971 (3)	1.1677 (2)	0.79486 (17)	0.0231 (5)	
H14	0.7773	1.2464	0.8256	0.028*	
C15	0.6700 (3)	1.1474 (2)	0.76767 (17)	0.0212 (5)	
H15	0.5634	1.213	0.7762	0.025*	
C16	0.7027 (3)	1.0283 (2)	0.72745 (16)	0.0180 (5)	
H16	0.6155	1.0126	0.7098	0.022*	
C17	0.2700 (3)	0.0954 (2)	0.98358 (19)	0.0329 (6)	
H17A	0.3251	0.0748	0.9105	0.049*	
H17B	0.2791	0.0131	1.0286	0.049*	
H17C	0.3244	0.1485	1.0036	0.049*	
C18	0.0950 (4)	0.1690 (3)	0.99553 (19)	0.0324 (6)	
N1	0.7503 (2)	0.70242 (16)	0.80927 (13)	0.0147 (4)	
N2	0.8527 (2)	0.93454 (16)	0.71257 (13)	0.0139 (4)	
N3	−0.0409 (4)	0.2285 (3)	1.0044 (2)	0.0611 (8)	
N4	0.3300 (2)	0.15790 (18)	0.65069 (14)	0.0209 (4)	
N5	0.5198 (2)	0.52975 (17)	0.72843 (14)	0.0188 (4)	
O1	0.5078 (2)	0.61883 (15)	0.65457 (12)	0.0275 (4)	
O2	0.65264 (19)	0.48347 (14)	0.75472 (12)	0.0234 (4)	
O1W	0.6855 (2)	0.79702 (17)	0.60155 (13)	0.0223 (4)	
O3	0.3987 (2)	0.48851 (19)	0.77325 (13)	0.0399 (5)	
O4	0.4269 (2)	0.22261 (17)	0.60683 (13)	0.0333 (4)	
O5	0.2323 (3)	0.1471 (2)	0.60526 (14)	0.0507 (6)	
O6	0.3220 (2)	0.10636 (19)	0.74130 (12)	0.0373 (5)	
S1	1.02040 (7)	0.53473 (5)	0.62853 (4)	0.01695 (13)	
S2	1.08663 (7)	0.81202 (5)	0.51967 (4)	0.01743 (13)	
Cu1	0.88876 (3)	0.75583 (2)	0.667678 (18)	0.01329 (9)	
H1D	0.696 (3)	0.815 (2)	0.545 (2)	0.02*	
H1E	0.650 (3)	0.739 (2)	0.6110 (19)	0.02*	

### Atomic displacement parameters (Å^2^)

**Table:**

	*U*^11^	*U*^22^	*U*^33^	*U*^12^	*U*^13^	*U*^23^
C1	0.0170 (12)	0.0159 (11)	0.0193 (11)	−0.0044 (9)	−0.0038 (9)	−0.0015 (9)
C2	0.0150 (11)	0.0211 (12)	0.0215 (12)	−0.0058 (10)	0.0007 (9)	−0.0030 (9)
C3	0.0239 (13)	0.0236 (12)	0.0160 (11)	−0.0113 (10)	0.0029 (9)	−0.0046 (9)
C4	0.0247 (13)	0.0191 (11)	0.0142 (11)	−0.0090 (10)	−0.0052 (9)	0.0012 (9)
C5	0.0205 (11)	0.0156 (11)	0.0131 (10)	−0.0068 (9)	−0.0050 (9)	−0.0026 (8)
C6	0.0180 (11)	0.0198 (11)	0.0151 (11)	−0.0071 (9)	−0.0065 (9)	0.0032 (9)
C7	0.0196 (12)	0.0167 (11)	0.0174 (11)	−0.0042 (10)	−0.0031 (9)	0.0017 (9)
C8	0.0157 (11)	0.0186 (11)	0.0210 (12)	−0.0040 (9)	0.0011 (9)	−0.0047 (9)
C9	0.0244 (13)	0.0239 (12)	0.0148 (11)	−0.0086 (10)	0.0024 (9)	−0.0078 (9)
C10	0.0142 (11)	0.0188 (11)	0.0208 (12)	−0.0071 (9)	−0.0018 (9)	−0.0020 (9)
C11	0.0140 (11)	0.0170 (11)	0.0218 (11)	−0.0046 (9)	−0.0058 (9)	−0.0014 (9)
C12	0.0159 (11)	0.0183 (11)	0.0120 (10)	−0.0077 (9)	−0.0023 (8)	0.0024 (8)
C13	0.0207 (12)	0.0226 (12)	0.0183 (11)	−0.0113 (10)	−0.0017 (9)	−0.0034 (9)
C14	0.0250 (13)	0.0169 (11)	0.0239 (12)	−0.0092 (10)	0.0023 (10)	−0.0041 (9)
C15	0.0182 (12)	0.0164 (11)	0.0210 (12)	−0.0016 (10)	−0.0001 (9)	0.0008 (9)
C16	0.0139 (11)	0.0186 (11)	0.0198 (11)	−0.0056 (9)	−0.0041 (9)	0.0026 (9)
C17	0.0342 (15)	0.0361 (14)	0.0242 (13)	−0.0051 (12)	−0.0078 (11)	−0.0044 (11)
C18	0.0366 (16)	0.0307 (14)	0.0284 (14)	−0.0107 (13)	−0.0067 (12)	−0.0025 (11)
N1	0.0167 (10)	0.0147 (9)	0.0123 (9)	−0.0060 (8)	−0.0023 (7)	−0.0010 (7)
N2	0.0134 (9)	0.0147 (9)	0.0129 (9)	−0.0051 (7)	−0.0030 (7)	0.0015 (7)
N3	0.0369 (16)	0.0621 (18)	0.074 (2)	−0.0040 (14)	−0.0147 (14)	−0.0030 (15)
N4	0.0180 (10)	0.0246 (10)	0.0180 (10)	−0.0055 (9)	−0.0026 (8)	−0.0029 (8)
N5	0.0207 (10)	0.0196 (10)	0.0158 (9)	−0.0068 (8)	−0.0019 (8)	−0.0052 (8)
O1	0.0310 (10)	0.0262 (8)	0.0294 (9)	−0.0141 (8)	−0.0157 (8)	0.0123 (7)
O2	0.0190 (9)	0.0237 (8)	0.0277 (9)	−0.0018 (7)	−0.0114 (7)	−0.0029 (7)
O1W	0.0265 (9)	0.0306 (10)	0.0163 (8)	−0.0168 (8)	−0.0102 (7)	0.0058 (7)
O3	0.0311 (10)	0.0629 (13)	0.0322 (10)	−0.0318 (10)	−0.0073 (8)	0.0128 (9)
O4	0.0285 (10)	0.0412 (10)	0.0362 (10)	−0.0229 (9)	−0.0069 (8)	0.0032 (8)
O5	0.0662 (14)	0.0919 (16)	0.0228 (10)	−0.0612 (13)	−0.0203 (9)	0.0136 (10)
O6	0.0240 (9)	0.0706 (13)	0.0171 (9)	−0.0205 (9)	−0.0069 (7)	0.0111 (8)
S1	0.0190 (3)	0.0175 (3)	0.0142 (3)	−0.0070 (2)	−0.0022 (2)	−0.0026 (2)
S2	0.0174 (3)	0.0196 (3)	0.0139 (3)	−0.0070 (2)	−0.0022 (2)	0.0012 (2)
Cu1	0.01294 (14)	0.01486 (14)	0.01176 (14)	−0.00559 (11)	−0.00217 (10)	0.00008 (10)

### Geometric parameters (Å, °)

**Table:**

C1—N1	1.345 (3)	C11—H11B	0.99
C1—C2	1.376 (3)	C12—N2	1.353 (3)
C1—H1	0.95	C12—C13	1.387 (3)
C2—C3	1.382 (3)	C13—C14	1.381 (3)
C2—H2	0.95	C13—H13	0.95
C3—C4	1.376 (3)	C14—C15	1.381 (3)
C3—H3	0.95	C14—H14	0.95
C4—C5	1.382 (3)	C15—C16	1.388 (3)
C4—H4	0.95	C15—H15	0.95
C5—N1	1.356 (2)	C16—N2	1.342 (3)
C5—C6	1.498 (3)	C16—H16	0.95
C6—C7	1.530 (3)	C17—C18	1.445 (4)
C6—H6A	0.99	C17—H17A	0.98
C6—H6B	0.99	C17—H17B	0.98
C7—S1	1.820 (2)	C17—H17C	0.98
C7—H7A	0.99	C18—N3	1.129 (3)
C7—H7B	0.99	N1—Cu1	2.0265 (17)
C8—C9	1.517 (3)	N2—Cu1	2.0169 (17)
C8—S1	1.826 (2)	N4—O4	1.233 (2)
C8—H8A	0.99	N4—O6	1.244 (2)
C8—H8B	0.99	N4—O5	1.252 (2)
C9—S2	1.817 (2)	N5—O3	1.239 (2)
C9—H9A	0.99	N5—O2	1.246 (2)
C9—H9B	0.99	N5—O1	1.263 (2)
C10—C11	1.529 (3)	O1W—Cu1	2.1342 (16)
C10—S2	1.830 (2)	O1W—H1D	0.74 (2)
C10—H10A	0.99	O1W—H1E	0.77 (3)
C10—H10B	0.99	S1—Cu1	2.3419 (6)
C11—C12	1.501 (3)	S2—Cu1	2.3488 (6)
C11—H11A	0.99		
			
N1—C1—C2	122.79 (19)	N2—C12—C11	116.83 (18)
N1—C1—H1	118.6	C13—C12—C11	122.20 (19)
C2—C1—H1	118.6	C14—C13—C12	119.4 (2)
C1—C2—C3	118.7 (2)	C14—C13—H13	120.3
C1—C2—H2	120.6	C12—C13—H13	120.3
C3—C2—H2	120.6	C15—C14—C13	119.8 (2)
C4—C3—C2	118.7 (2)	C15—C14—H14	120.1
C4—C3—H3	120.7	C13—C14—H14	120.1
C2—C3—H3	120.7	C14—C15—C16	117.9 (2)
C3—C4—C5	120.56 (19)	C14—C15—H15	121
C3—C4—H4	119.7	C16—C15—H15	121
C5—C4—H4	119.7	N2—C16—C15	122.6 (2)
N1—C5—C4	120.49 (19)	N2—C16—H16	118.7
N1—C5—C6	117.97 (18)	C15—C16—H16	118.7
C4—C5—C6	121.51 (18)	C18—C17—H17A	109.5
C5—C6—C7	113.22 (17)	C18—C17—H17B	109.5
C5—C6—H6A	108.9	H17A—C17—H17B	109.5
C7—C6—H6A	108.9	C18—C17—H17C	109.5
C5—C6—H6B	108.9	H17A—C17—H17C	109.5
C7—C6—H6B	108.9	H17B—C17—H17C	109.5
H6A—C6—H6B	107.7	N3—C18—C17	178.7 (3)
C6—C7—S1	113.97 (14)	C1—N1—C5	118.68 (18)
C6—C7—H7A	108.8	C1—N1—Cu1	118.34 (13)
S1—C7—H7A	108.8	C5—N1—Cu1	122.85 (14)
C6—C7—H7B	108.8	C16—N2—C12	119.15 (18)
S1—C7—H7B	108.8	C16—N2—Cu1	121.40 (14)
H7A—C7—H7B	107.7	C12—N2—Cu1	119.34 (14)
C9—C8—S1	108.29 (15)	O4—N4—O6	121.63 (19)
C9—C8—H8A	110	O4—N4—O5	119.84 (18)
S1—C8—H8A	110	O6—N4—O5	118.48 (19)
C9—C8—H8B	110	O3—N5—O2	120.81 (18)
S1—C8—H8B	110	O3—N5—O1	119.09 (19)
H8A—C8—H8B	108.4	O2—N5—O1	120.10 (18)
C8—C9—S2	112.79 (15)	Cu1—O1W—H1D	121 (2)
C8—C9—H9A	109	Cu1—O1W—H1E	112.8 (18)
S2—C9—H9A	109	H1D—O1W—H1E	102 (3)
C8—C9—H9B	109	C7—S1—C8	101.14 (10)
S2—C9—H9B	109	C7—S1—Cu1	105.19 (7)
H9A—C9—H9B	107.8	C8—S1—Cu1	98.81 (7)
C11—C10—S2	114.91 (15)	C9—S2—C10	102.25 (10)
C11—C10—H10A	108.5	C9—S2—Cu1	102.29 (7)
S2—C10—H10A	108.5	C10—S2—Cu1	100.95 (7)
C11—C10—H10B	108.5	N2—Cu1—N1	92.23 (7)
S2—C10—H10B	108.5	N2—Cu1—O1W	101.61 (7)
H10A—C10—H10B	107.5	N1—Cu1—O1W	91.34 (7)
C12—C11—C10	111.75 (17)	N2—Cu1—S1	159.72 (5)
C12—C11—H11A	109.3	N1—Cu1—S1	91.39 (5)
C10—C11—H11A	109.3	O1W—Cu1—S1	98.25 (5)
C12—C11—H11B	109.3	N2—Cu1—S2	84.82 (5)
C10—C11—H11B	109.3	N1—Cu1—S2	169.43 (5)
H11A—C11—H11B	107.9	O1W—Cu1—S2	99.19 (5)
N2—C12—C13	120.82 (19)	S1—Cu1—S2	88.00 (2)
			
N1—C1—C2—C3	0.6 (3)	C11—C10—S2—C9	−111.41 (16)
C1—C2—C3—C4	−2.2 (3)	C11—C10—S2—Cu1	−6.11 (16)
C2—C3—C4—C5	1.4 (3)	C16—N2—Cu1—N1	−71.14 (15)
C3—C4—C5—N1	1.0 (3)	C12—N2—Cu1—N1	105.23 (15)
C3—C4—C5—C6	−176.8 (2)	C16—N2—Cu1—O1W	20.69 (16)
N1—C5—C6—C7	−70.0 (2)	C12—N2—Cu1—O1W	−162.94 (15)
C4—C5—C6—C7	107.9 (2)	C16—N2—Cu1—S1	−171.24 (11)
C5—C6—C7—S1	67.2 (2)	C12—N2—Cu1—S1	5.1 (3)
S1—C8—C9—S2	58.91 (18)	C16—N2—Cu1—S2	119.04 (15)
S2—C10—C11—C12	−60.7 (2)	C12—N2—Cu1—S2	−64.60 (14)
C10—C11—C12—N2	65.2 (2)	C1—N1—Cu1—N2	70.66 (16)
C10—C11—C12—C13	−110.3 (2)	C5—N1—Cu1—N2	−113.39 (16)
N2—C12—C13—C14	−3.1 (3)	C1—N1—Cu1—O1W	−31.02 (16)
C11—C12—C13—C14	172.21 (18)	C5—N1—Cu1—O1W	144.93 (16)
C12—C13—C14—C15	−0.8 (3)	C1—N1—Cu1—S1	−129.30 (15)
C13—C14—C15—C16	2.9 (3)	C5—N1—Cu1—S1	46.64 (15)
C14—C15—C16—N2	−1.2 (3)	C1—N1—Cu1—S2	144.2 (2)
C2—C1—N1—C5	1.8 (3)	C5—N1—Cu1—S2	−39.9 (4)
C2—C1—N1—Cu1	177.89 (16)	C7—S1—Cu1—N2	62.99 (16)
C4—C5—N1—C1	−2.5 (3)	C8—S1—Cu1—N2	−41.17 (16)
C6—C5—N1—C1	175.32 (18)	C7—S1—Cu1—N1	−37.25 (9)
C4—C5—N1—Cu1	−178.48 (15)	C8—S1—Cu1—N1	−141.42 (9)
C6—C5—N1—Cu1	−0.6 (3)	C7—S1—Cu1—O1W	−128.81 (9)
C15—C16—N2—C12	−2.6 (3)	C8—S1—Cu1—O1W	127.03 (9)
C15—C16—N2—Cu1	173.80 (16)	C7—S1—Cu1—S2	132.19 (8)
C13—C12—N2—C16	4.7 (3)	C8—S1—Cu1—S2	28.02 (7)
C11—C12—N2—C16	−170.80 (17)	C9—S2—Cu1—N2	157.96 (9)
C13—C12—N2—Cu1	−171.73 (15)	C10—S2—Cu1—N2	52.70 (8)
C11—C12—N2—Cu1	12.8 (2)	C9—S2—Cu1—N1	83.8 (3)
C6—C7—S1—C8	96.11 (17)	C10—S2—Cu1—N1	−21.5 (3)
C6—C7—S1—Cu1	−6.32 (17)	C9—S2—Cu1—O1W	−101.09 (9)
C9—C8—S1—C7	−161.96 (15)	C10—S2—Cu1—O1W	153.65 (9)
C9—C8—S1—Cu1	−54.46 (15)	C9—S2—Cu1—S1	−3.05 (8)
C8—C9—S2—C10	73.74 (18)	C10—S2—Cu1—S1	−108.31 (7)
C8—C9—S2—Cu1	−30.51 (17)		

### Hydrogen-bond geometry (Å, °)

**Table:**

*D*—H···*A*	*D*—H	H···*A*	*D*···*A*	*D*—H···*A*
O1W—H1D···O5^i^	0.74 (3)	1.94 (3)	2.669 (2)	169 (3)
O1W—H1E···O1	0.77 (2)	2.00 (3)	2.754 (3)	166 (2)
C3—H3···O2^ii^	0.95	2.55	3.487 (3)	168
C8—H8A···O1^iii^	0.99	2.54	3.447 (3)	152
C8—H8B···O4^iii^	0.99	2.55	3.341 (3)	137
C10—H10A···O1^iii^	0.99	2.43	3.228 (3)	137
C10—H10B···O5^iv^	0.99	2.45	3.271 (3)	140
C13—H13···O6^iv^	0.95	2.42	3.277 (3)	149
C14—H14···O2^v^	0.95	2.56	3.220 (3)	127
C16—H16···O6^v^	0.95	2.36	3.126 (3)	137
C17—H17A···O6	0.98	2.26	3.145 (3)	150

Symmetry codes: (i) −*x*+1, −*y*+1, −*z*+1; (ii) −*x*+1, −*y*+1, −*z*+2; (iii) *x*+1, *y*, *z*; (iv) *x*+1, *y*+1, *z*; (v) *x*, *y*+1, *z*.
